# Baltimore Academy of Medicine

**Published:** 1886-04

**Authors:** 


### THE BALTIMORE ACADEMY OF MEDICINE.

Officially Reported for the ATLANTA MEDICAL AND SURGICAL JOURNAL.

STATED MEETING, Held February 16, 1886.

After the regular order of business, Dr. A. B. Arnold related an interesting case of

RAYNOD'S DISEASE (?)

that had recently come under his notice; it occurred in a man, a stoker on a steamboat. The patient presented the following lesions: From the first finger of his right hand the last two phalanges had disappeared, and from the ring finger of the same hand the last phalanx had dropped off. On the left hand the last phalanx of the thumb was gone, and the last phalanx of the little finger had loosened preparatory to spontaneous amputation. He considered this a case identical with three described by Raynod, all of whose cases occurred in men following the same occupation (stokers) and all having lost portions of their fingers in manner similar to Dr. Arnold's man.

The pathology of the affection is very obscure. The patients complain of no pain whatever, and as a rule are in a tolerably good general condition.

Around about the stump, in Dr. Arnold's case, everything was perfectly normal, not so much as a dry eczema being present. The only other lesion that he could detect on the man's body was an ugly excoriation in the palm of the left hand, which he did not think bore any relation to the special condition referred to.

He had seen another case with a similar loss of portions of the fingers, but the condition about the stump suggested elephantiasis.

Dr. J. Edwin Michael said he thought he had seen the same case, as the man had been making the rounds of the different hospitals in the city. It was his impression that the same case had been reported sometime since, in the *Journal of Cutaneous and Venereal Diseases*, by Dr. Robert B. Morison, of this city. He said when he saw him that the lesion in the palm of the left hand, to which Dr. Arnold had referred, communicated with a sinus which ran for some distance up the forearm and from which pus could be squeezed, but when the patient left the hospital the palmar wound had entirely healed.

Dr. J. J. Chisolm had seen one of these cases some years since, but took it to be elephantiasis.

Dr. Michael agreed with Dr. Arnold in not thinking it of syphilitic origin.

Dr. Arnold said, in answer to Dr. Chisolm's question, "How does it differ from leprosy?" that there is no anæsthesia, no false œdema, etc.

Dr. Henry M. Wilson wished to know if leprosy was not on the increase in this country.

Dr. Arnold said that recently several cases of true tuberculous, anæsthetic leprosy had been imported into this country and had been reported.

Dr. J. J. Chisolm said he recalled five cases of true tuberculous leprosy, two of which occurred in persons with whom he was personally acquainted. These cases he had seen while engaged in practice of medicine in South Carolina. They occurred in natives, whose parents were also born in this country. They all occurred in white males.

Dr. J. Edwin Michael referred to a case reported by Dr. I. E. Atkinson, which existed and had its origin in Baltimore city. It was in a white woman. This, too, was true tuberculous leprosy.

Dr. Frank Donaldson, Jr., then read his admission thesis entitled

DIAPHRAGMATIC PLEURISY.

It will be published in the April number of the *Interna'ional Journal of Medical Sciences.*

URETHAN.

Dr. John N. Uhler called the attention of the Academy to the new hypnotic, urethan. He gets most favorable results from its use. It is not disagreeable of taste, causes pleasant, refreshing sleep, and is devoid both of nauseating and bad after-effects.

He gives it in doses ranging from five grains to twenry-five grains, fifteen grains being the average. He substitutes it very effectually for chloral, pareldehyde and opium. He has not used it as an anodyne, but only as an hypnotic. Considers its effects intermediate between chloral and pareldehyde.

Dr. J. J. Chisolm referred to a case that illustrates the rapidity of restoration to sight after operation in cases of complete glaucoma. In this case vision was perfect after ten days.

Dr. Samuel Theobald referred to some interesting results he had recently obtained by the use of salicylate of sodium in iritis, occurring in patients with a history of inflammatory rheumatism.

Three weeks ago a young lady had called to see him—she had a most intense plastic iritis—pupil perfectly immovable. She had only perception of light, and could not count fingers at the ordinary distance. She was ordered sodium salicylate and in a few days she obtained much relief. This salt was then withdrawn and iodide of iron ordered. She did well until the end of several days, when she reappeared complaining of the same trouble, which increased in intensity until at the end of two days she was in the same condition as originally. The salicylate was repeated as well as the atropia drops, locally, and mercurial inunctions to temples. Cathartics were ordered, and in *two days* she was so very much improved that vision equalled $\frac{20}{64}$ and the media sufficiently clear to permit satisfactory inspection of the optic disk.

He had had some glaucomatous tension, but this did not prevent his ordering atropia, nor interfere with the physiological and therapeutical action of the drug.

Dr. J. J. Chisolm has had some astonishing results in these cases from large doses of salicylate of sodium. In choroiditis he gets remarkable results from the use of pilocarpine. Two decided sweats give pronounced relief.

Dr. H. P. C. Wilson spoke of a case in which he was recently credited with being the cause of the death of one of his patients. The woman was from a malarious district, much run down, etc. She came to him to be operated on for laceration of cervix uteri.

After treating her with tonics, etc., until her general condition was improved, he decided to operate and appointed the hour; shortly before the appointed time arrived, the patient was taken with colicy pains in the abdomen consequent upon drinking a glass of milk; these pains were allayed by the ordinary measures, and she then insisted upon his proceeding with the operation.

Local anæsthesia was obtained by the use of cocaine and the operation performed without accident. After completion of the operation, the pains returned and were allayed by hypodermic injections of morphia. She spent a very restless night, and when he visited her the next morning found her quite ill.

Later in the day an erysipelatous inflammation appeared in the skin of the external ear and became so intense that the patient succumbed on the next day. There was nothing whatever wrong at the seat of operation.

